# A “Tuned” Mask Learnt Approach Based on Gravitational Search Algorithm

**DOI:** 10.1155/2016/8179670

**Published:** 2016-12-19

**Authors:** Youchuan Wan, Mingwei Wang, Zhiwei Ye, Xudong Lai

**Affiliations:** ^1^School of Remote Sensing and Information Engineering, Wuhan University, Wuhan 430072, China; ^2^School of Computer Science, Hubei University of Technology, Wuhan 430068, China

## Abstract

Texture image classification is an important topic in many applications in machine vision and image analysis. Texture feature extracted from the original texture image by using “Tuned” mask is one of the simplest and most effective methods. However, hill climbing based training methods could not acquire the satisfying mask at a time; on the other hand, some commonly used evolutionary algorithms like genetic algorithm (GA) and particle swarm optimization (PSO) easily fall into the local optimum. A novel approach for texture image classification exemplified with recognition of residential area is detailed in the paper. In the proposed approach, “Tuned” mask is viewed as a constrained optimization problem and the optimal “Tuned” mask is acquired by maximizing the texture energy via a newly proposed gravitational search algorithm (GSA). The optimal “Tuned” mask is achieved through the convergence of GSA. The proposed approach has been, respectively, tested on some public texture and remote sensing images. The results are then compared with that of GA, PSO, honey-bee mating optimization (HBMO), and artificial immune algorithm (AIA). Moreover, feature extracted by Gabor wavelet is also utilized to make a further comparison. Experimental results show that the proposed method is robust and adaptive and exhibits better performance than other methods involved in the paper in terms of fitness value and classification accuracy.

## 1. Introduction

Texture [1] is an important characteristic of the appearance of objects in natural scenes and is a powerful visual cue, used by both humans and machines in describing and recognizing objects of the real world. Texture image classification [2] is a vital topic in machine vision and image analysis, which is to identify a texture sample as one of several possible classes with a reliable texture classifier, and plays a very important role in a wide range of applications. In the real world, there are kinds of texture due to changes in orientation, scale, or other visual appearance; as a result, a number of texture feature extraction and classification methods have been proposed over the years. For instance, Xu et al. [3] developed a novel tool called dynamic fractal analysis for dynamic texture (DT) classification, which not only provided a rich description of DT but also had strong robustness to environmental changes. Liu et al. [4] presented a simple, novel, and yet very powerful approach for robust rotation-invariant texture classification based on random projection, which maintained the strengths of random projection, in being computationally efficient and low-dimensional. Celik and Tjahjadi [5] proposed a supervised multiscale Bayesian texture classifier by obtaining complex-valued multiscale representations of training texture samples for each texture class. Zhang et al. [6] utilized the normalized local-oriented energies to generate the local feature vectors, which described the local structures distinctively and are less sensitive to imaging conditions. Thakare and Patil [7] presented an improved method for texture image classification and retrieval using gray level cooccurrence matrix (GLCM) and self-organizing maps (SOM). Riaz et al. [8] and Li et al. [9] introduced a novel technique to rotation and scale invariant texture classification based on Gabor wavelet feature that have the capability to collapse the filter responses according to the scale and orientation of the texture features. Liu et al. [10] and Zhao et al. [11] presented a novel approach for texture feature classification by generalizing the well-known local binary pattern (LBP) approach. The experimental results showed that the proposed method was robust to noise and could achieve impressive classification accuracy. Gai et al. [12] and Soulard Carré [13] presented a study of the wavelet transform (WT) which had one shift invariant magnitude and three angle phases at each scale from texture image analysis application. The experimental results demonstrated the robustness of the proposed method and obtained satisfied accuracy. Texture feature especially is one of the most significant symbols for remote sensing image classification. For instance, residential area is one of the most important landscape elements. Extraction of residential area by remote sensing image has become the favored technique to monitor urban expansion and environment, which is significant to the regional sustainable development. Some studies have been focused on the field of residential area recognition by texture feature; for example, information of residential area was extracted by airborne SAR aided with gray level cooccurrence matrix (GLCM) texture feature [14]. Wang et al. [15] proposed a Gabor filtering based method to recognize residential areas from remotely sensed imagery. Jin et al. [16] presented a residential area recognition method for some remote sensing images based on Fourier transformation and Hough transformation. Shi et al. [17] proposed an extended oscillatory correlation algorithm to perform unsupervised scene recognition of residential areas for hyperspectral imagery. Experiment demonstrated the utility of the proposed method for residential areas recognition. However, it expends numerous features to complete the task of texture feature classification for some traditional techniques, which needs a large amount of CPU time to extract the features, and the excessive features will decrease the classification efficiency at the same time. Although there are some methods that only need a few of features, it is difficult to stably obtain high classification accuracy.

In order to extract the texture feature efficiently and effectively, the texture feature classification technique based on texture mask has drawn rather considerable interest in recent years [18]. Among them, Laws' mask [19] is one of the most commonly used masks to classify the different types of texture. However, the basic form of Laws' mask is relatively stationary, which is difficult to adapt various types of texture for a fixed mask [20]. Thus, You and Cohen [21] developed an adaptive texture feature extraction method called “Tuned” mask exempted from changes in rotation and scale of the texture image and its validity was proved. To obtain the optimal texture mask, it utilized a search strategy of gradient estimation and random search with heuristic learning. It may lead to high time complexity and probably trap into the local optimum [21].

In essence, how to obtain the optimal texture mask is a combinatorial optimization problem which may be handled by evolutionary algorithms and swarm intelligence algorithms. For instance, Zheng et al. [22] proposed a mask approach optimized by artificial immune algorithm (AIA) to detect texture objects on satellite images. H. Zheng and Z. Zheng [23] employed genetic algorithm (GA) guided search to obtain optimal “Tuned” mask and produced rather good results. Ye et al. [24] explained the principle and steps of producing texture “Tuned” mask with particle swarm optimization algorithm (PSO) and illustrated how to train “Tuned” mask with the proposed method in details. Zheng [25] introduced a honey-bee model and provided a new method of producing better “Tuned” mask with honey-bee mating optimization (HBMO), which was applied to texture classification of aerial images. The experiments showed that the proposed method could improve the quality of “Tuned” mask and classification accuracy. In short, AIA, GA, PSO, and HBMO could obtain good “Tuned” mask; however, it is a very hard optimization problem with high dimension, and the value of each dimension might be a real number in the range of wide continuous space; that is, algorithms mentioned above could not guarantee the optimal solution; it is worth trying more evolutionary algorithms and swarm intelligence based algorithms on this topic.

Gravitational search algorithm (GSA) [26] is a newly proposed stochastic global search algorithm. Nowadays, GSA has been widely used in diverse applications; for example, Yazdani et al. [27] utilized GSA to find multiple solutions in multimodal problems. Kumar and Sahoo [28] presented the compendious survey on the GSA and its applications as well as enlightened the applicability of GSA in data clustering and classification. Duman et al. [29] used GSA to find the solution for optimal power flow (OPF) problem in a power system. In the field of classification, GSA was used to provide a prototype classifier to face the classification of instances in multiclass datasets [30]. Sarafrazi and Nezamabadi-pour [31] hybridized GSA with support vector machine (SVM) and made a novel GSA-SVM hybrid system to improve classification accuracy in binary problems. Further, there are some variants and modifications of GSA; for example, Rashedi et al. [32] proposed a binary coded GSA (BGSA) and used it for benchmark functions. A modified GSA with moving strategy was utilized to solve the problem of path planning of uninhabited aerial vehicle (UAV) [33]. Li and Duan [34] proposed a chaotic GSA (CGSA) for the parameter identification problem of chaotic system, which performed better than the standard GSA. However, the standard GSA is by far the most popularly used, and the optimal “Tuned” mask is a combinatorial optimization problem, which could be solved by GSA. Hence, in this paper, a novel residential areas recognition technique is proposed using “Tuned” mask and blending of standard GSA.

The rest of this paper is structured as follows.  Section 2 illustrates the basic principle of gravitational search algorithm. The idea of the proposed approach to produce the optimal “Tuned” mask is detailed in Section 3.  Section 4 displays the experimental results and discussion. Finally, the paper is concluded in Section 5.

## 2. The Basic Principle of Gravitational Search Algorithm

In 2009, Rashedi et al. have developed a new swarm intelligence algorithm named gravitational search algorithm (GSA) though Newtonian laws of gravity and mass interaction, which has the enormous potential to solve the combinatorial optimization problem [26]. In this algorithm, agents are considered as objects and their performance is evaluated by their masses. Each object is a solution for the problem. Objects will be mutually attracted by the gravity force, and the force leads to a global movement of all objects to which have heavier masses [35]. Because the heavier masses could have good solutions, they are more likely to obtain the optimal solution and they move sluggishly than lighter masses that represent worse solutions. In GSA, there are four particulars for every mass: position, inertial mass, active gravitational mass, and passive gravitational mass [35]. The position represents one of the solutions of the problem and the gravitational and inertial masses are utilized as a fitness function.

Assume that there is a system with *N* agents (objects); the position of *i*th agent can be defined as

(1) $$\begin{array}{c} X_{i}=\left(\;x_{i}^{1},\ldots ,x_{i}^{d},\ldots ,x_{i}^{n}\;\right),\;i=1,2,\ldots ,N, \end{array}$$

where *x*_*i*_^*d*^ represents the position of *i*th object in the *d*th dimension and *n* is the dimension of search space. According to the theory of GSA, the gravitational force between the object *i* and *j* at iteration *t* could be defined by

(2) $$\begin{array}{c} F_{ij}^{d}\left(\;t\;\right)=G\left(\;t\;\right)\frac{M_{pi}\left(t\right)\times M_{aj}\left(t\right)}{R_{ij}\left(t\right)+\varepsilon }\left(\;x_{j}^{d}\left(\;t\;\right)-x_{i}^{d}\left(\;t\;\right)\;\right), \end{array}$$

where *M*_*aj*_ is the active gravitational mass of object *j*, *M*_*pi*_ is the passive gravitational mass correlated with object *i*, *ε* is a small constant, *R*_*ij*_(*t*) is the Euclidian distance from the object *i* to object *j* at iteration *t*, and *G*(*t*) is gravitational variable at iteration *t*, which could be defined as

(3) $$\begin{array}{c} G\left(\;t\;\right)=G_{0}e^{-\alpha \left(t/T\right)}, \end{array}$$

where *G*_0_ is the initial value of *G*(*t*), *α* is a constant by manual setting, *t* is the current iteration, and *T* is the maximum iteration number.

Moreover, the total gravitational force acting that works on *i*th object is a randomly weighted sum of *d*th component of the forces, which is computed as

(4) $$\begin{array}{c} F_{i}^{d}\left(\;t\;\right)=\underset{j\in Kbest,j\neq i}{\sum }{rand}_{j}F_{ij}^{d}\left(\;t\;\right), \end{array}$$

where rand_*j*_ is a uniform random variable in the interval [0,1] and *K*best is the set of first *K* agents with the optimal fitness value and biggest mass, which is a function related to time and is initialized as *K*_0_ at the beginning and decreased with iteration.

By the law of motion, the acceleration of *i*th object at iteration *t* and in direction *d* is calculated as follows:

(5) $$\begin{array}{c} a_{i}^{d}\left(\;t\;\right)=\frac{F_{i}^{d}\left(t\right)}{M_{ii}\left(t\right)}, \end{array}$$

where *M*_*ii*_ is the inertial mass of *i*th object.

The velocity at iteration *t* + 1 of an object is considered as an addition of the velocity and acceleration at iteration *t*. Therefore, the new velocity *v*_*i*_^*d*^(*t* + 1) and position *x*_*i*_^*d*^(*t* + 1) at iteration *t* + 1 could be calculated as follows:

(6) vidt+1=randi×vidt+aidt,xidt+1=xidt+vidt+1,

where rand_*i*_ is a random number within interval [0,1].

Gravitational and inertia masses are simply computed by the fitness value. A heavier mass means a good solution, which means that the better object has higher attractions and walks more sluggishly. Suppose that the gravitational and inertia mass are equalized, the values of masses are calculated using the map of fitness. The gravitational and inertial masses will be updated by the following equations:

(7) Mai=Mpi=Mii=Mi,i=1,2,…,N,qit=fitit−worsttbestt−worstt,Mit=qit∑j=1Nqjt,

where fit_*i*_(*t*) is the fitness value of the object *i* at iteration *t* and worst(*t*) and best(*t*) should be defined as follows (for a minimization problem):

(8) worstt=maxj∈1,…,N fitjt,

(9) bestt=minj∈1,…,N fitjt.

It is clear that, for a maximization problem, (8) and (9) will be replaced by (10) and (11), respectively:

(10) worstt=minj∈1,…,N fitjt,

(11) bestt=maxj∈1,…,N fitjt.

As GSA is applied to solve the combinatorial optimization problem, each object is located at a certain position of the search space, which represents a solution of the problem at each iteration. Then, the objects will be updated and the next positions and velocities are calculated by (6). Other parameters of GSA like the gravitational variable *G*, the active gravitational mass *M*_*ai*_, the passive gravitational mass *M*_*pi*_, the inertial mass *M*_*ii*_, and the acceleration *a*_*i*_^*d*^ will be, respectively, computed by other equations. The basic procedures of GSA could be described as in Pseudocode 1 [26].

## 3. The Proposed Method

In this section, an efficient texture feature classification method with “Tuned” mask is expounded, which learn the parameters of “Tuned” mask as a combinatorial optimization problem by using GSA. The goal of the proposed method is to maximize the classification accuracy by only one feature. The main procedure of the proposed method will be explained as follows.

### 3.1. The Fundamental of “Tuned” Mask

In order to utilize the optimal texture mask and make an accurate classification for different texture features, You and Cohen [21] suggested the extension of Laws' scheme by abandoning the traditional masks with constants and replacing them with variables in order to improve the classification accuracy and reliability. In the method, a single 5 × 5 mask is produced which extracts a common feature of a single texture at different rotations and scales; at the same time, it discriminates this feature from other texture features to a large extent. The new mask is called a “Tuned” or adaptive mask, and the whole process of texture feature classification is very simple. In principle, the procedure to capture texture characterization comprises two steps. The first step is to convolve the whole image with the “Tuned” mask *A*. Experimental results showed that the mask with symmetrical and zero sums will reduce the computation cost, which nearly does not have effect on the performance of the mask [23]. Thus, the whole mask could be composed by only 10 parameters. The 2D convolution of the original image *I*(*m*, *n*) with size *M* × *N* and mask *A*(*m*, *n*) with size (2*a* + 1) × (2*a* + 1) is computed as below:

(12) Fm,nAm,n∗Im,n=∑k=−ak=a ∑l=−al=aAm,n·Im+k,n+l,

where “*∗*” represents convolution operation and “·” represents the multiplication operation, *F*(*m*, *n*) is the image after transformation, *k* and *l* are, respectively, the translation variable of horizontal and vertical, and *a* is a constant as *a* = 2 in the paper.

The second step is to make a statistics within *w*_*x*_ × *w*_*y*_ (9 × 9 is used in the paper) window at pixel point (*m*, *n*). The “texture energy” TE could be calculated by the variance statistic within macro window size of 9 × 9 in our training stage, which is defined as [36]

(13) TE=∑wx∑wyFm,n2P2×wx×wy,

(14) P2=∑i,jAm,n2.

It is apparent that the value of texture energy is decided by mask; the optimal “Tuned” mask could provide favorable criminating ability. In this paper, the newly proposed evolutionary algorithm GSA is employed to generate the robust “Tuned” mask and make classification for different textural images.

### 3.2. The Encoding Schema

The key issue to apply GSA is the representation of the problem, that is, how to make a suitable mapping between the problem solution and each agent (object) of GSA. In the paper, a search space for a mask is of 25 dimensions. Each dimension has continuous or integer values. H. Zheng and Z. Zheng suggested employing the symmetrical mask with zero sums to avoid plenty of computation [23]. Therefore, the “Tuned” mask could be defined as below:

(15) $$\begin{array}{c} {mask}_{i}=\left[\begin{array}{ccccc} x_{i}^{1} & x_{i}^{2} & -2\left(x_{i}^{1}+x_{i}^{2}\right) & x_{i}^{2} & x_{i}^{1}\\ x_{i}^{3} & x_{i}^{4} & -2\left(x_{i}^{3}+x_{i}^{4}\right) & x_{i}^{4} & x_{i}^{3}\\ x_{i}^{5} & x_{i}^{6} & -2\left(x_{i}^{5}+x_{i}^{6}\right) & x_{i}^{6} & x_{i}^{5}\\ x_{i}^{7} & x_{i}^{8} & -2\left(x_{i}^{7}+x_{i}^{8}\right) & x_{i}^{8} & x_{i}^{7}\\ x_{i}^{9} & x_{i}^{10} & -2\left(x_{i}^{9}+x_{i}^{10}\right) & x_{i}^{10} & x_{i}^{9} \end{array}\right]. \end{array}$$

As the size of “Tuned” mask is 5 × 5 and requires being symmetrical with zero sums, so only 10 parameters *x*_*i*_^1^,  *x*_*i*_^2^,  *x*_*i*_^3^,  *x*_*i*_^4^,  *x*_*i*_^5^,  *x*_*i*_^6^,  *x*_*i*_^7^,  *x*_*i*_^8^,  *x*_*i*_^9^,  *x*_*i*_^10^ in a mask need to be encoded. In a “Tuned” mask, the layout of parameters in the mask plays a more important role for texture image classification than its actual values. Due to the fact that the decimal code can be directly used for GSA, the parameters of *x*_*i*_^1^,  *x*_*i*_^2^,  *x*_*i*_^3^,  *x*_*i*_^4^,  *x*_*i*_^5^,  *x*_*i*_^6^,  *x*_*i*_^7^,  *x*_*i*_^8^,  *x*_*i*_^9^,  *x*_*i*_^10^ are encoded by decimal number in the range of [−50, 50] for simplicity [23].

### 3.3. The Objective Function

In order to make an evaluation for the optimization ability of GSA and other evolutionary algorithms, it is necessary to choose a suitable objective function. Due to the fact that the residential areas' recognition could be considered as a binary-class classification problem, this regards residential areas as a category and other texture areas as another category. Fisher's criterion has a good performance for binary-class classification problem, which tries to maximize the difference of interclass and minimize the difference of intraclass and precisely recognize the target category from another category [37]. Therefore, in the paper, the objective function within Fisher's criterion is defined as

(16) $$\begin{array}{c} fit=\frac{{\left(\mu_{1}-\mu_{2}\right)}^{2}}{\sigma_{1}^{2}+\sigma_{2}^{2}}, \end{array}$$

where *μ*_1_ and *σ*_1_^2^ are, respectively, the average and variance of the eigenvalues in the first category and *μ*_2_ and *σ*_2_^2^ are, respectively, the average and variance of the eigenvalues in the second category. The larger value of the fitness function demonstrates better quality of “Tuned” mask.

### 3.4. Implementation of the Proposed Method

The proposed method is simple and easy to implement. The main process to learn the “Tuned” mask based on GSA for texture feature classification is as in Pseudocode 2.

## 4. Simulation Results and Discussion

The proposed method is implemented by the language of MATLAB 2014b on a personal computer with a 2.30 GHz CPU, 8.00 G RAM under Windows 8 system.

In order to evaluate the performance of the proposed residential areas' recognition method, 3 texture images from public texture database and 5 remote sensing images are, respectively, used in this section. The objective function is defined as (16). A higher fitness value of fitness function indicates better optimization ability.

To make a fair comparison, the number of function evaluations is used as terminal criterion; that is, all algorithms will stop when the number of function evaluations reaches 1000, and all the algorithms make 50 independent operations. In the section, we present some contrastive experimental results, including illustrative examples and performance evaluating tables, which clearly demonstrate the merits of the proposed method. All the algorithms are evaluated using the same objective function. Our primary interest is the optimal “Tuned” mask, which is shown by the fitness value of objective function defined as (16), and the classification accuracy by using the optimal “Tuned” mask.

### 4.1. Parameters Setting for Different Algorithms

According to the operational process of evolutionary computation algorithm, the computational results of GSA depend on parameters setting to some extent; fine tuning of the parameters can produce a better result.  Table 1 shows the parameters used in GSA.

Some commonly used evolutionary algorithm or swarm intelligence based texture feature classification methods are also carried out for comparison in the paper as well. As is illustrated in Section 2, the primary GSA is used in this paper. Some existing “Tuned” mask techniques which are, respectively, proposed by Zheng (GA [23], HBMO [25]) and Ye et al. (PSO [24]) are used to make a comparison. On the other hand, Zheng et al. utilized another texture energy function to detect texture objects [22], and experimental results demonstrated the validity, so Zheng's mask [22] was used and, respectively, optimized by AIA and GSA in this paper. Furthermore, the commonly used Gabor wavelet feature [8, 9] is also utilized to make a comparison, which totally includes 56 features (7 scales and 8 orientations) here. Although there are many variants of GA, PSO, AIA, and HBMO, in order to make a fair comparison, GA, PSO, AIA, and HBMO are all used with their standard types. Tables 2 [Table tab3] [Table tab4]–5 show the parameters setting of GA [38], PSO [39], AIA [40], and HBMO [41].

### 4.2. Experiments on Public Texture Images

**Figure 1:**
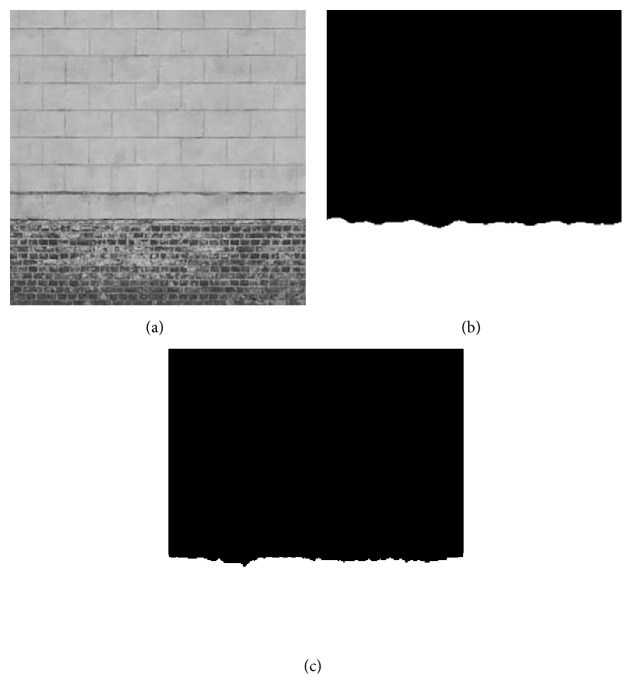
Recognition of Brick image: (a) original image, (b) recognized result of Gabor wavelet, and (c) recognized result of proposed method.

**Figure 2 fig2:**
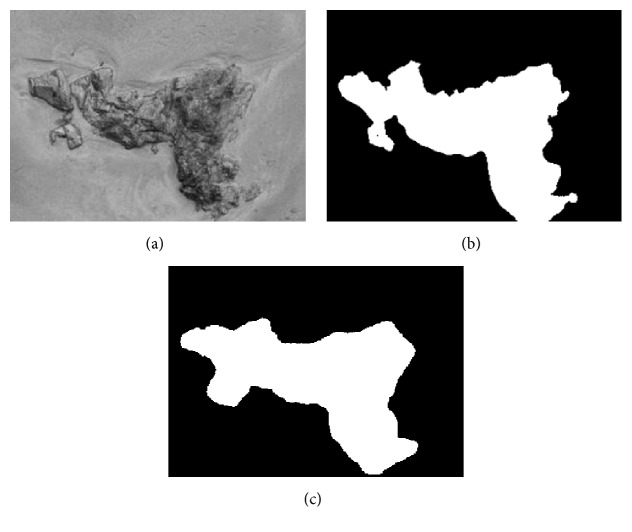
Recognition of Rock image: (a) original image (b) recognized result of Gabor wavelet (c) recognized result of proposed method.

**Figure 3:**
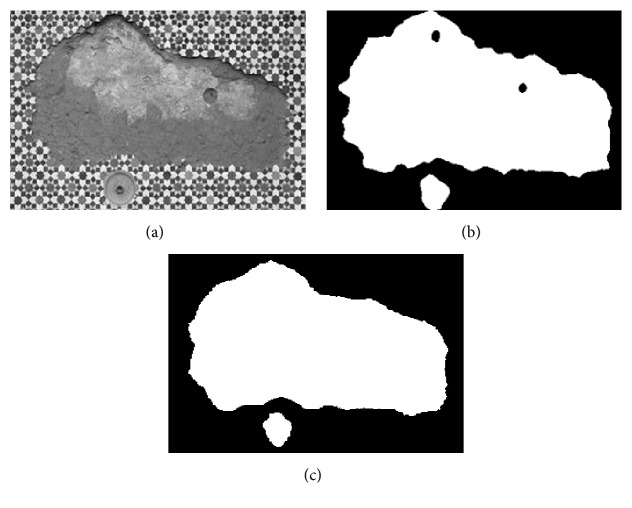
Recognition of Tile image: (a) original image, (b) recognized result of Gabor wavelet, and (c) recognized result of proposed method.

Here, a preliminary test of the proposed texture feature classification technique on 3 texture images, respectively, named “Brick,” “Rock,” and “Tile” from a public texture database (http://www.textures.com) is conducted, and the images' sizes are, respectively, 380 × 380, 350 × 250, and 337 × 227. 30 training samples are utilized for classification, the size is 50 × 50, and all training samples are all extracted from the original image. As the optimal “Tuned” mask is achieved, the classification for each pixel of the original image is accomplished by using the minimum distance classifier.  Table 6 shows the fitness value and classification accuracy of the “Tuned” mask handled by different algorithms. Furthermore, Table 7 shows the classification accuracy by using Zheng's mask and Gabor wavelet based feature, and the testing images and recognized result will be given in Figures 1 [Fig fig2]–3.

In Tables 6 and 7, Avg and Std, respectively, indicate the average and the standard deviation of the fitness value by making 50 independent operations. Accuracy is the average classification accuracy of 50 independent operations. Time is the CPU time of each iteration; its unit is second. “Tuned”-AIA and “Tuned”-GSA denote the recognition result by using “Tuned” mask that is optimized by AIA and GSA. Zheng-AIA and Zheng-GSA denote the recognized result by using Zheng's mask optimized by AIA and GSA. Gabor indicates the recognized result by using Gabor wavelet feature. According to the data in Table 6, the classification accuracy is close for all algorithms; the maximum difference is less than 3%, and for “Tile” image, the difference is only 1.6%. However, GSA still has the best optimization ability in five algorithms, its average fitness value is the maximum for the 3 images, and the average classification accuracy has exceeded 92%; although the average fitness value of GSA and HBMO is very similar, the standard deviation of fitness value by using GSA is the minimum for 3 images, which proves that GSA can more stably converge to the optimal solution. For computation efficiency, PSO and GSA have a fast convergence speed comparing with the other three algorithms; the maximum difference of CPU time between them is less than 0.03 s for each iteration, but the fitness value by using GSA is obviously better than PSO; the average fitness value by using GSA is more than 29 for 3 images. According to Figures 1 [Fig fig2]–3, although the features based on Gabor wavelet could make a rough recognition for the object, the edge selection is distinctly worse than that by using the proposed method. In Table 7, it is evidently revealed that Zheng's mask [22] will cost more time, and the classification accuracy is distinctly lower than that by using “Tuned” mask, and the difference has reached 5% for “Brick” and “Tile” images. Consequently, it may deduce that the proposed method can be widely used to make recognition for different texture areas.

### 4.3. Experiments on Remote Sensing Images

**Figure 4:**
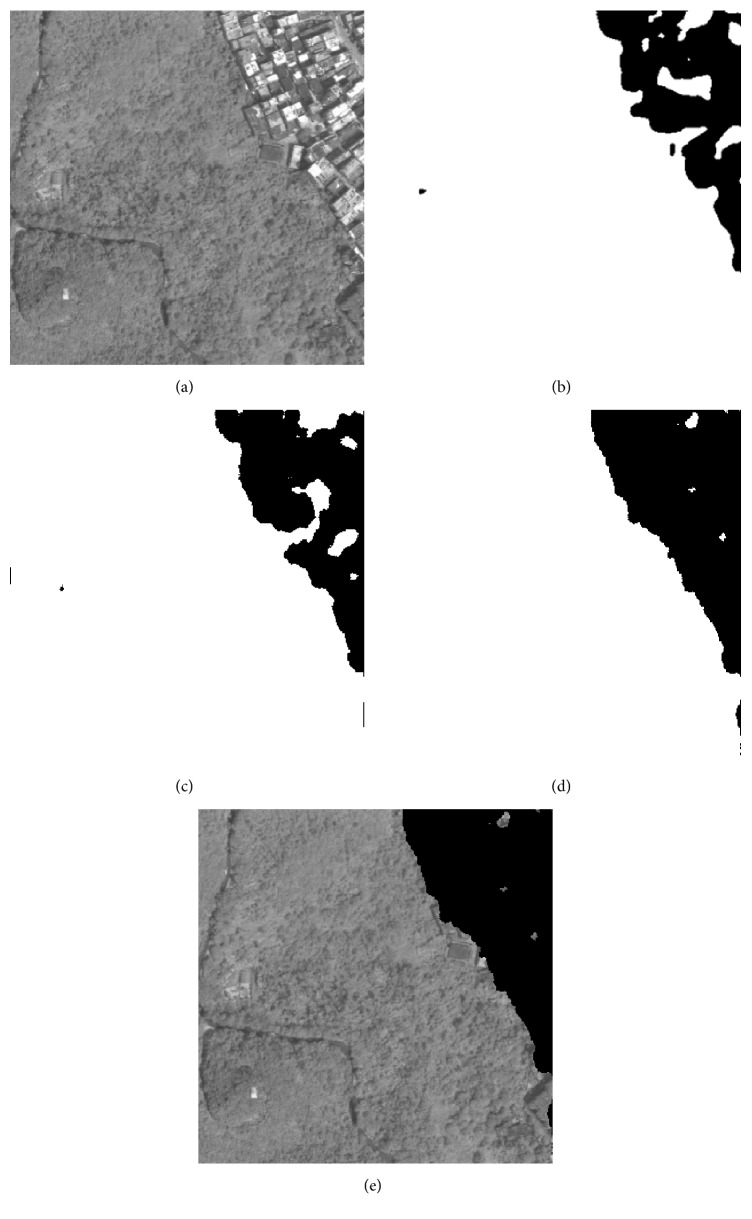
Recognition result of RS1 image: (a) original image, (b) recognized result of Gabor wavelet, (c) recognized result of Zheng's mask, (d) recognized result of proposed method, and (e) superimposition image.

**Figure 5 fig5:**
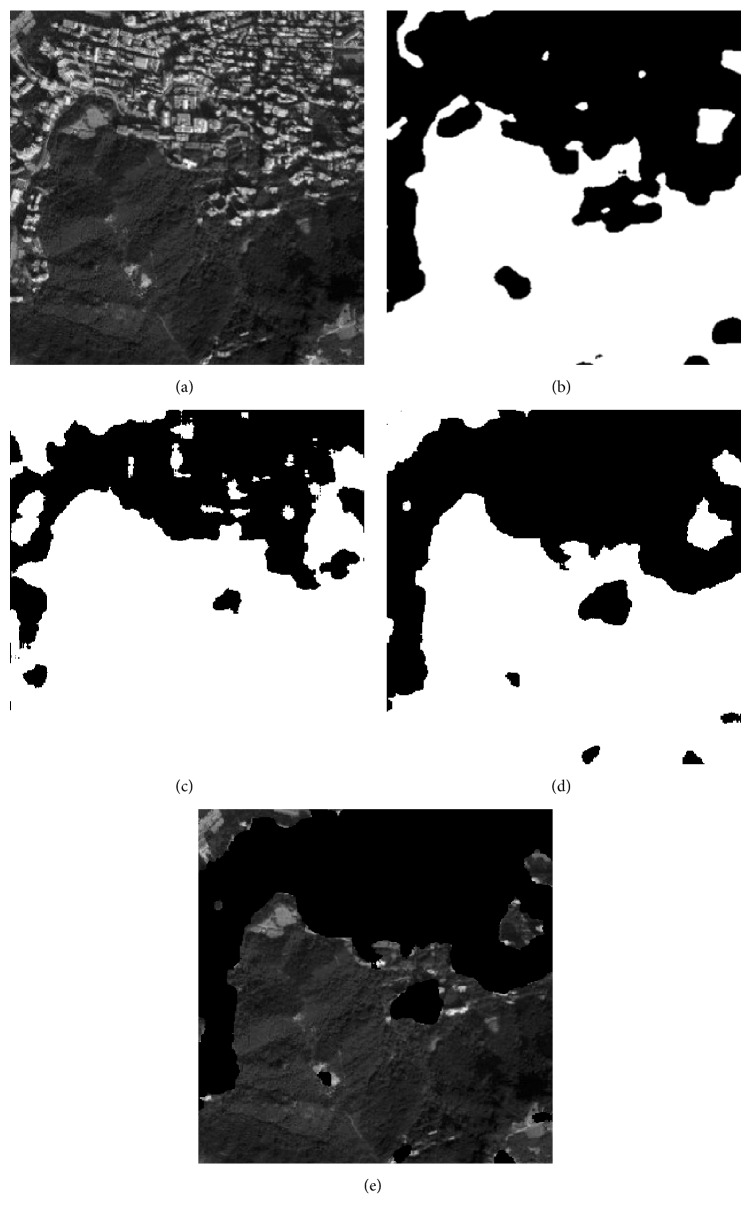
Recognition of RS2 image: (a) original image, (b) recognized result of Gabor wavelet, (c) recognized result of Zheng's mask, (d) recognized result of proposed method, and (e) superimposition image.

**Figure 6 fig6:**
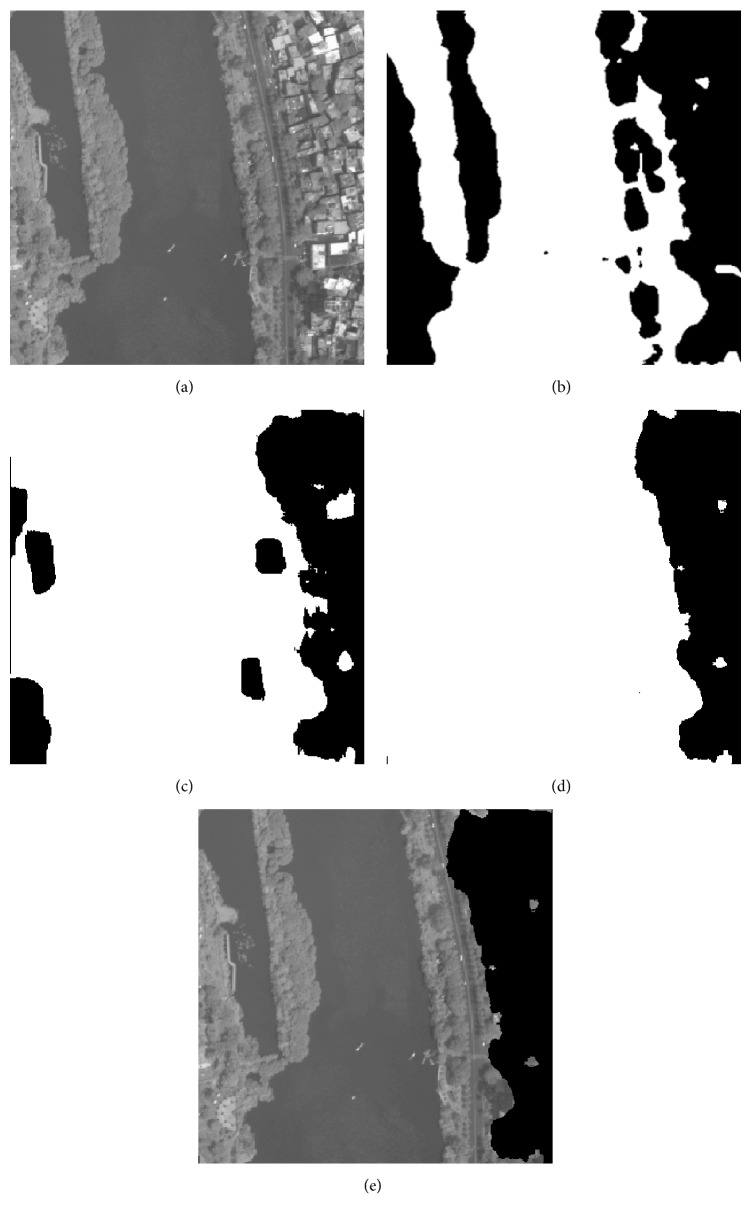
Recognition of RS3 image: (a) original image, (b) recognized result of Gabor wavelet, (c) recognized result of Zheng's mask, (d) recognized result of proposed method, and (e) superimposition image.

**Figure 7 fig7:**
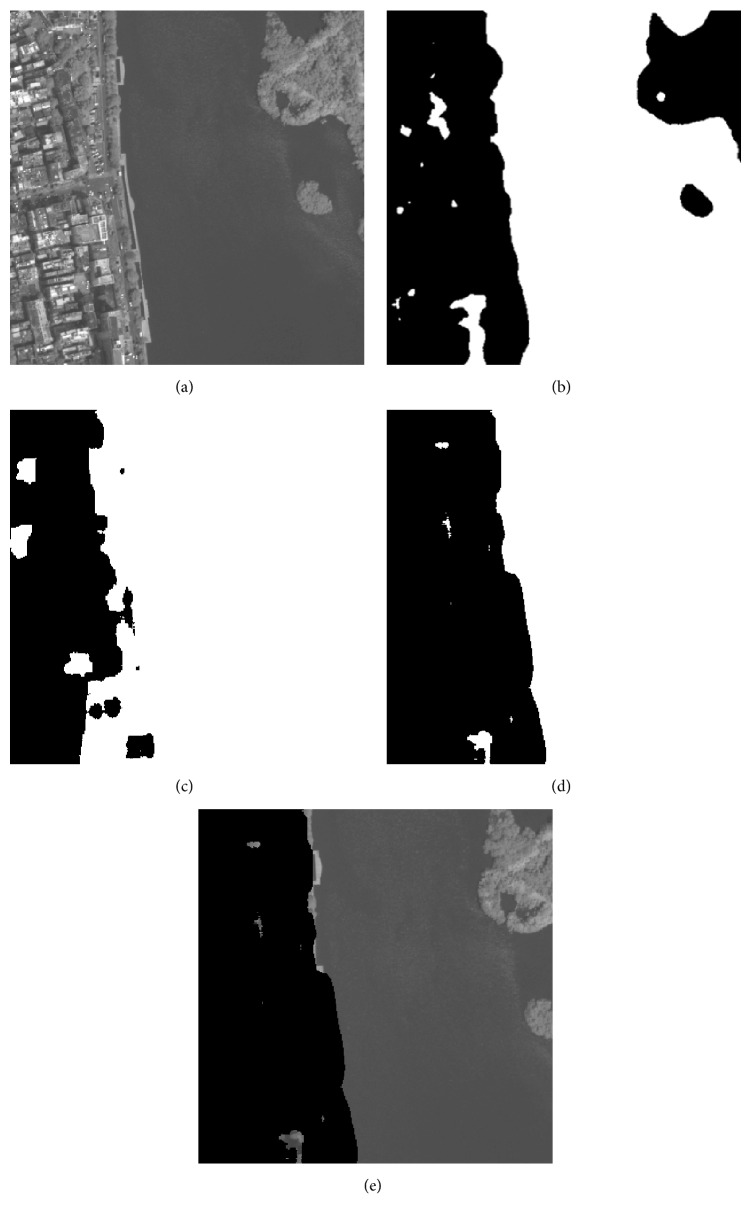
Recognition of RS4 image: (a) original image, (b) recognized result of Gabor wavelet, (c) recognized result of Zheng's mask, (d) recognized result of proposed method, and (e) superimposition image.

**Figure 8:**
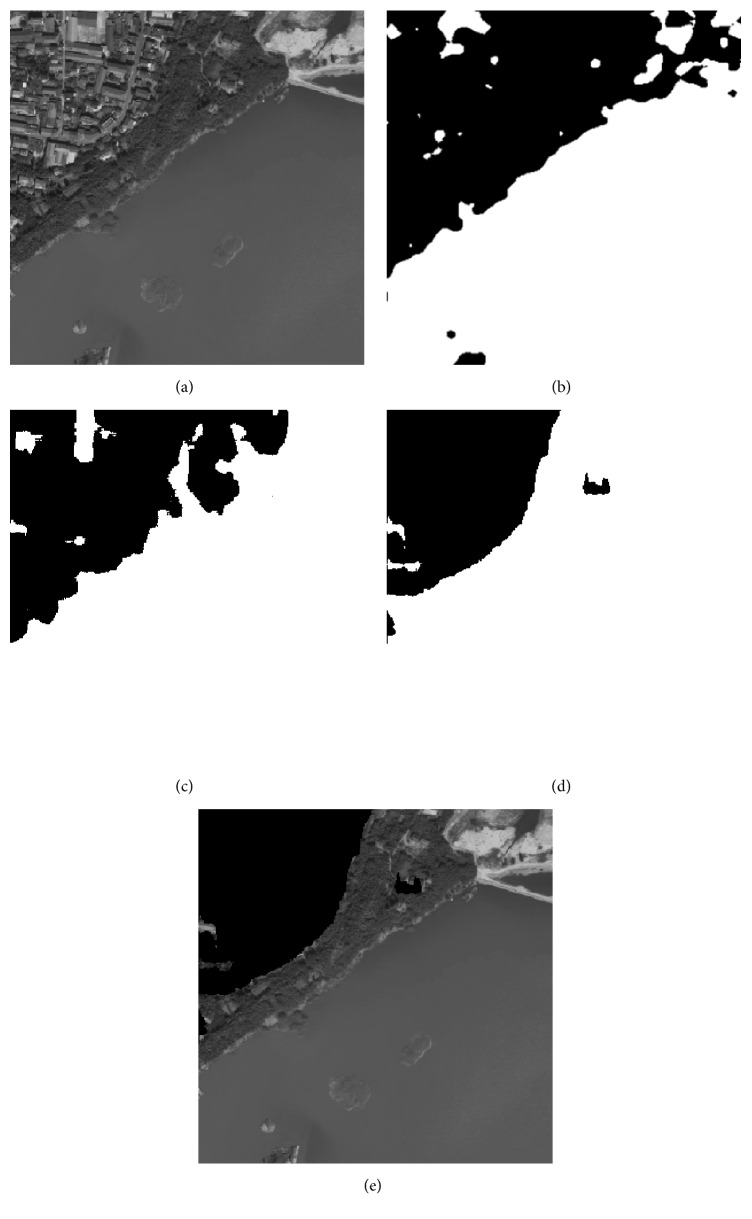
Recognition of RS5 image: (a) original image, (b) recognized result of Gabor wavelet, (c) recognized result of Zheng's mask, (d) recognized result of proposed method, and (e) superimposition image.

As it is illustrated in Section 4.2, the proposed method has good classification result for public texture dataset, which manifests that it is suitable for texture feature classification. In this section, 5 remote sensing images that include part of residential areas, respectively, named RS1, RS2, RS3, RS4, and RS5 are utilized to make a further experiment, and the images' sizes are all 400 × 400. The training samples are all extracted from the original image. As the optimal “Tuned” mask is achieved, the classification for each pixel of the original image is accomplished by using the minimum distance classifier.  Table 8 shows the fitness value and classification accuracy of the “Tuned” mask optimized by different algorithms.  Table 9 shows the classification accuracy with Zheng's mask and Gabor wavelet based feature, and the recognized result will be given in Figures 4 [Fig fig5] [Fig fig6] [Fig fig7]–8.

In Tables 8 and 9, Avg and Std, respectively, indicate the average and the standard deviation of the fitness value by making 50 independent operations. Accuracy is the average classification accuracy of 50 independent operations. Time is the CPU time at each iteration; its unit is second. The meanings of “Tuned”-AIA, “Tuned”-GSA, Zheng-AIA, Zheng-GSA and Gabor are the same as that of Table 7. As the texture feature of remote sensing images is more random, the fitness value for RS3, RS4, and RS5 images is obviously lower than other images; recognition of residential area is more complex, thus, it is easy to misidentify the objective areas. According to the data in Table 8, GSA could obtain the maximum average fitness value, and, for RS2 image, the average fitness of GSA is more than 49, which illustrate that the optimization ability of GSA has a distinct advantage comparing with the other 4 algorithms, and the recognition ability of different categories is apparent. On the other hand, the standard deviation of fitness value by using GSA is the minimum; and for RS3, RS4, and RS5 images, the standard deviation of fitness value is all less than 0.1, which is a small range and nearly has no volatility; and it is illustrated that the algorithm could stably converge to a satisfied solution for each independent experiment. The classification accuracy by using GSA has exceeded 86% for 5 images, and it has reached 97.1932% for RS1 image especially, which is a satisfied accuracy for practical application, and the residential areas have been generally recognized. For converge efficiency, it is the same with the last section that PSO and GSA can quickly converge to the optimal solution, and the difference is only 0.08 s for each iteration. However, the fitness value by using GSA is distinctly better than PSO; the maximum difference of average fitness value between them has reached 1.5. Meanwhile, the advantage of mask technique is better than that of Gabor wavelet based method; in addition, the classification result of Zheng's mask [22] is only 72.9157% and 69.6710% for RS4 and RS5 images, which is apparently worse than that by using “Tuned” mask. More importantly, “Tuned” mask will cost fewer CPU time at the same time. Experiment results demonstrate that GSA has a better optimization ability comparing with other 4 algorithms, and “Tuned” mask is a feasible approach for texture feature classification, which only needs fewer parameters, and has satisfactory classification accuracy; particularly for residential area recognition, the classification accuracy is apparently better than Zheng's mask [22].

## 5. Conclusion

In conclusion, a residential area recognition method based on “Tuned” mask and optimized with gravitational search algorithm (GSA) is detailed. Three texture images from public texture database and 5 remote sensing images are used to make an evaluation for the proposed method. Results are compared with some other mask based classification techniques optimized by GA, PSO, AIA, and HBMO. In general, it is observed that evolutionary algorithm and swarm intelligence algorithm can be well used to complete the task of texture feature classification. Among these algorithms, GSA has a better performance; the average fitness value is higher than the other 4 algorithms; that is, GSA is more appropriate to be employed to obtain the optimal “Tuned” mask than GA, PSO, AIA, and HBMO. Moreover, in terms of CPU time, GSA can quickly converge to the optimal solution, which is quite fast enough to meet real-time applications. On the other hand, in order to make a more comprehensive comparison, features based on Gabor wavelet and another mask technique which is proposed by Zheng et al. [22] are also used in this paper and the mask is optimized by AIA and GSA. It is revealed that the proposed method has a better performance; the classification accuracy is satisfied. In sum, “Tuned” mask has a stable performance for texture feature classification in most cases. Further, the disadvantage of heavy computation efficiency could be conquered at the maximum degree when it is combined with GSA. The proposed method is able to keep a good balance between the efficiency and classification accuracy, which makes it more suitable for some texture feature classification applications.

## Figures and Tables

**Pseudocode 1:**
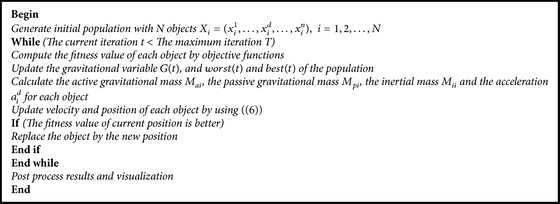
Pseudocode of GSA.

**Pseudocode 2:**
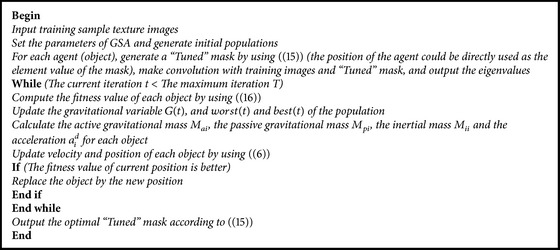
Pseudocode of learning the “Tuned” mask based on GSA.

**Table 1:** Parameters used in GSA.

Parameter	Explanation	Value
*N*	Number of agents (objects)	20
*G*_0_	Initial value of the gravitational variable *G*(*t*)	100
*α*	User specified constant	10

**Table 2:** Parameters used in GA.

Parameter	Explanation	Value
*N*	Number of genetics	20
*P*_*s*_	Selection ratio	0.9
*P*_*c*_	Crossover ratio	0.8
*P*_*m*_	Mutation ratio	0.01

**Table 3 tab3:** Parameters used in PSO.

Parameter	Explanation	Value
*N*	Number of particles	20
*c*_1_, *c*_2_	Positive acceleration constants	2.0
*r*_1_, *r*_2_	Random numbers	[0,1]

**Table 4 tab4:** Parameters used in AIA.

Parameter	Explanation	Value
*N*	Number of antibodies	20
*B*	Antibody elimination rate	0.3
*P*_*c*_	Crossover ratio	0.8
*P*_*m*_	Mutation ratio	0.01

**Table 5:** Parameters used in HBMO.

Parameter	Explanation	Value
	Number of queens	1
*N*_Drone_	Number of drones	20
*N*_Brood_	Number of broods	10
*α*	Decreasing factor	0.98

**Table 6:** Result of different algorithms for public texture images.

Dataset	Meas.	GA	PSO	AIA	HBMO	GSA
Brick	Avg	28.8825	30.4275	31.5182	31.7380	32.6939
	Std	2.2992	1.4374	0.8639	0.8022	0.6479
	Accuracy (%)	93.4493	94.5665	95.3433	95.7774	96.3742
	Time	0.2753	0.2728	0.2780	0.2782	0.2746

Rock	Avg	27.0690	27.9645	28.8741	28.9564	29.4108
	Std	1.7956	0.8883	0.8296	0.7970	0.6602
	Accuracy (%)	89.0483	90.1657	91.0628	91.3764	92.0350
	Time	0.2767	0.2735	0.2794	0.2910	0.2759

Tile	Avg	48.0013	48.7289	49.4354	49.5988	50.1986
	Std	2.4916	1.8612	1.5016	1.3950	1.0025
	Accuracy (%)	93.6011	94.1028	94.5532	94.7149	95.0289
	Time	0.2814	0.2755	0.2847	0.2948	0.2783

**Table 7:** Result of different methods for public texture images.

Dataset	Meas.	“Tuned”-AIA	“Tuned”-GSA	Zheng-AIA	Zheng-GSA	Gabor
Brick	Accuracy (%)	95.3433	96.3742	88.2992	91.3456	90.9904
	Time	0.2780	0.2746	0.3061	0.2947	1.3642

Rock	Accuracy (%)	91.0628	92.0350	83.7705	87.1574	86.2962
	Time	0.2794	0.2759	0.3084	0.2977	1.3851

Tile	Accuracy (%)	94.5532	95.0289	86.9648	89.2396	88.4899
	Time	0.2847	0.2783	0.3152	0.2998	1.4661

**Table 8:** Result of different algorithms for remote sensing images.

Dataset	Meas.	GA	PSO	AIA	HBMO	GSA
RS1	Avg	34.2731	36.7696	36.7657	37.0125	38.0333
	Std	5.1505	4.2538	4.4363	3.0899	2.2603
	Accuracy (%)	93.9797	95.4275	95.1400	96.0333	97.1932
	Time	0.2779	0.2652	0.2793	0.2915	0.2729

RS2	Avg	46.9603	47.4975	48.3295	48.4530	49.0828
	Std	1.4241	0.9470	0.8591	0.8315	0.4004
	Accuracy (%)	93.4484	94.2963	95.1594	95.5927	96.2379
	Time	0.2770	0.2639	0.2782	0.2897	0.2719

RS3	Avg	5.3701	5.4712	5.5026	5.5160	5.5837
	Std	0.1162	0.0883	0.0702	0.0639	0.0506
	Accuracy (%)	85.1405	87.3292	88.4858	89.1554	90.7826
	Time	0.2763	0.2634	0.2775	0.2865	0.2700

RS4	Avg	6.9304	7.0397	7.0858	7.1006	7.1720
	Std	0.1520	0.1177	0.0960	0.0926	0.0855
	Accuracy (%)	82.4348	83.9884	84.7993	85.2154	86.6071
	Time	0.2768	0.2638	0.2780	0.2893	0.2717

RS5	Avg	4.3903	4.5223	4.7030	4.6988	4.8958
	Std	0.1463	0.1307	0.1274	0.1144	0.0963
	Accuracy (%)	83.1671	85.0394	87.6705	87.1144	89.9387
	Time	0.2762	0.2631	0.2771	0.2861	0.2698

**Table 9:** Result of different methods for remote sensing images.

Dataset	Meas.	“Tuned”-AIA	“Tuned”-GSA	Zheng-AIA	Zheng-GSA	Gabor
RS1	Accuracy (%)	95.1400	97.1932	84.7560	90.2846	85.6994
	Time	0.2793	0.2729	0.3040	0.2920	1.4166

RS2	Accuracy (%)	95.1594	96.2379	83.0675	88.1354	84.9965
	Time	0.2782	0.2719	0.3030	0.2914	1.3696

RS3	Accuracy (%)	88.5848	90.7836	78.5296	81.4144	75.1974
	Time	0.2775	0.2700	0.3014	0.2902	1.2956

RS4	Accuracy (%)	84.7993	86.6071	72.9157	79.3633	74.0210
	Time	0.2780	0.2717	0.3032	0.2917	1.3554

RS5	Accuracy (%)	87.6705	89.9378	69.6710	76.5925	72.4010
	Time	0.2771	0.2698	0.2995	0.2876	1.2829
